# Supplementary material for: An Individualized Multi-Modal Approach for Detection of Medication “Off” Episodes in Parkinson’s Disease via Wearable Sensors
Source: J Pers Med. 2023 Jan 31;13(2):265. doi: 10.3390/jpm13020265 (PMC9962500; doi:10.3390/jpm13020265)
Supplement: Supplementary file 1 [file jpm-13-00265-s001.zip › jpm-2132922-supplementary.pdf]

Journal of *Personalized Medicine*

Supplementary Materials

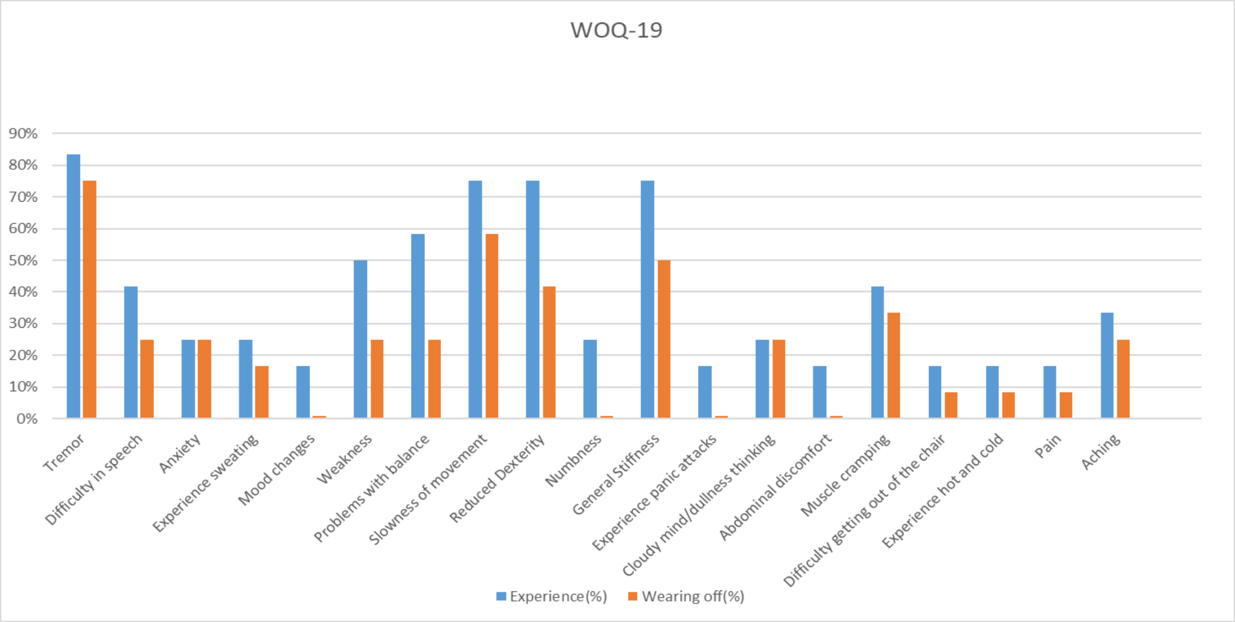

Figure S1. Percentage of experienced and wearing-off symptoms assessed through WOQ-19.

(A)

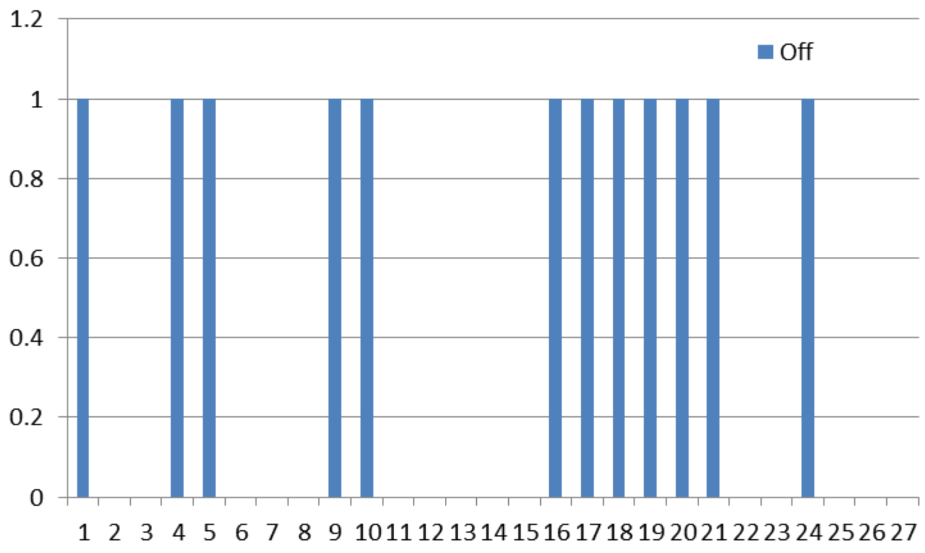

(B)

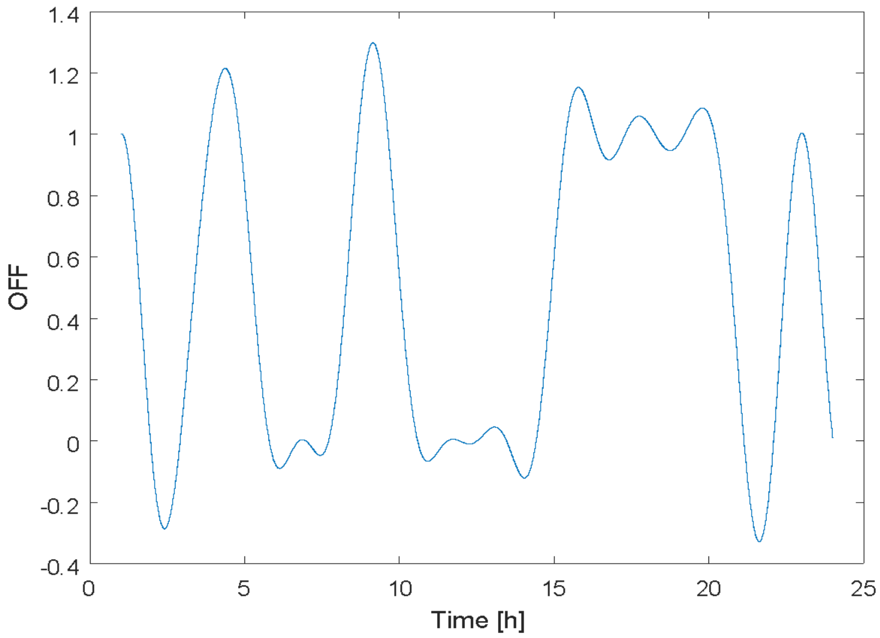

Figure S2. OFF state of the first subject. (A) The 24-element reported vector of OFF state. (B) Resampled OFF signal.

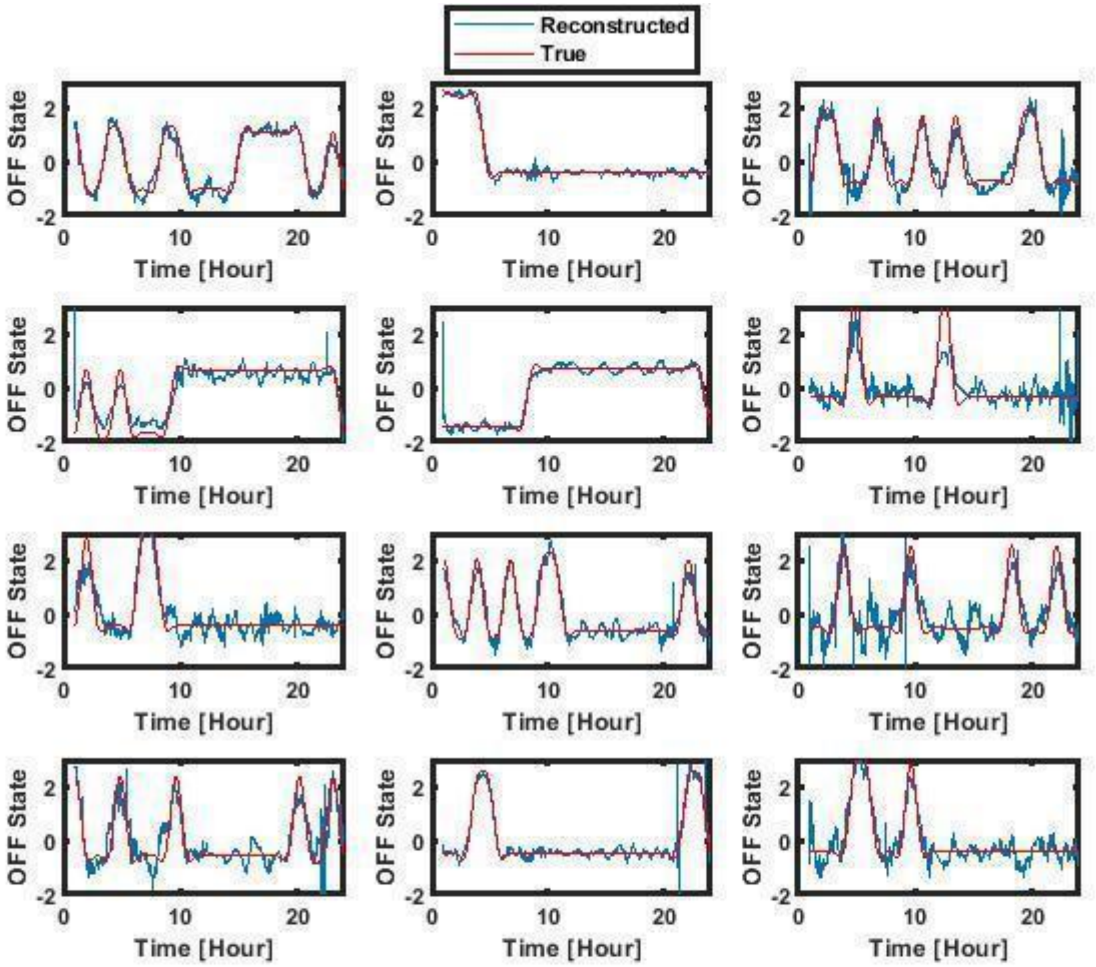

Figure S3. Reconstructed vs. true (centralized) OFF states for each subject. .

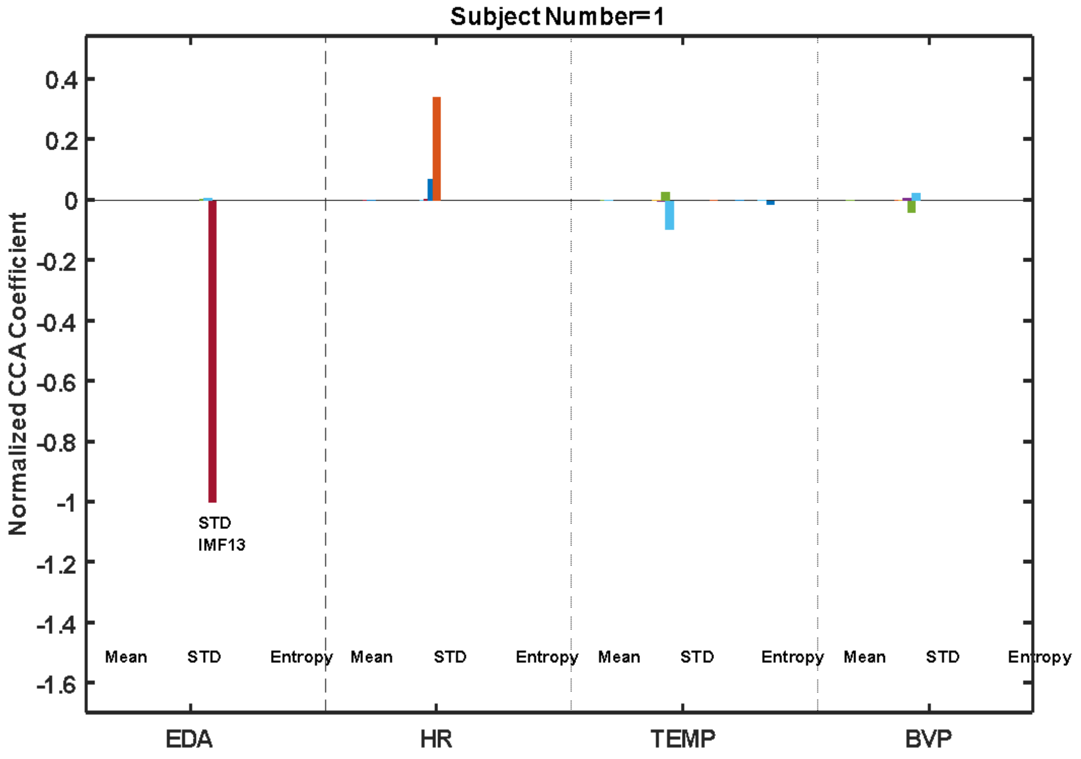

Subject Number=1
Normalized CCA Coefficient
0.4
0.2
0
-0.2
-0.4
-0.6
-0.8
-1
-1.2
-1.4
-1.6
STD
IMF13
Mean STD Entropy Mean STD Entropy Mean STD Entropy Mean STD Entropy
EDA
HR
TEMP
BVP

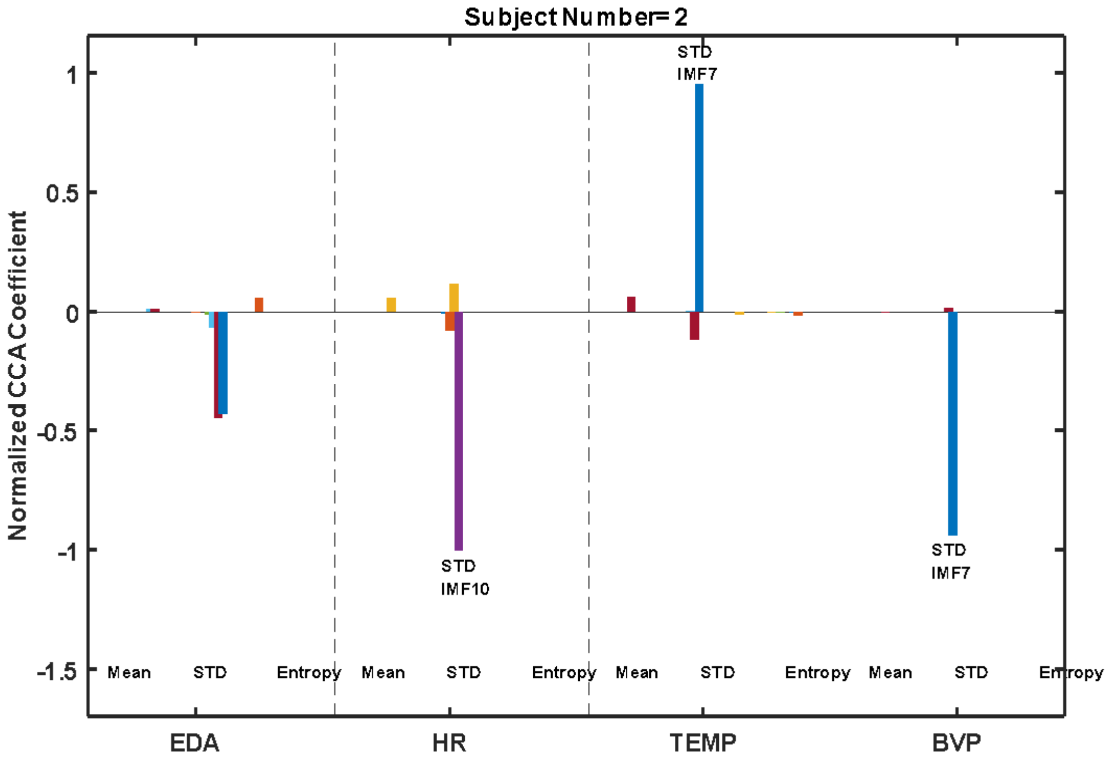

Subject Number=2
Normalized CCA Coefficient
1
0.5
0
-0.5
-1
-1.5
STD
IMF7
STD
IMF10
STD
IMF7
Mean STD Entropy Mean STD Entropy Mean STD Entropy Mean STD Entropy
EDA
HR
TEMP
BVP

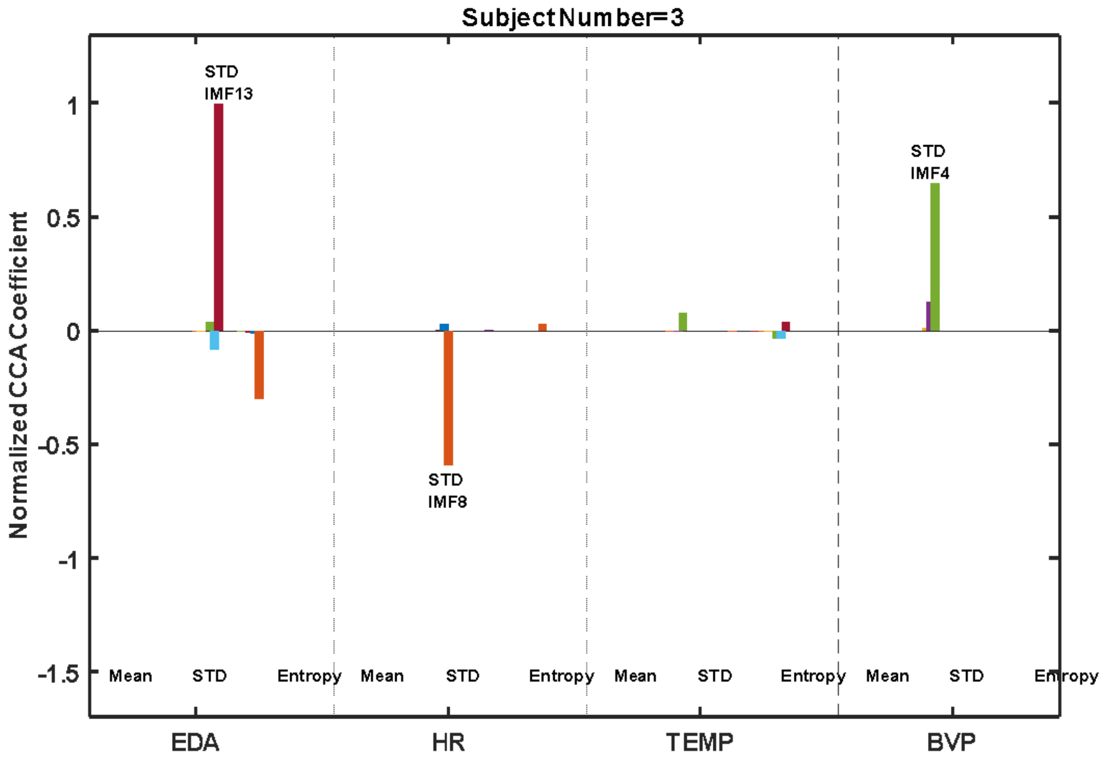
Subject Number=3
STD
IMF13
STD
IMF4
STD
IMF8
Normalized CCA Coefficient
1
0.5
0
-0.5
-1
-1.5
Mean STD Entropy Mean STD Entropy Mean STD Entropy Mean STD Entropy
EDA
HR
TEMP
BVP

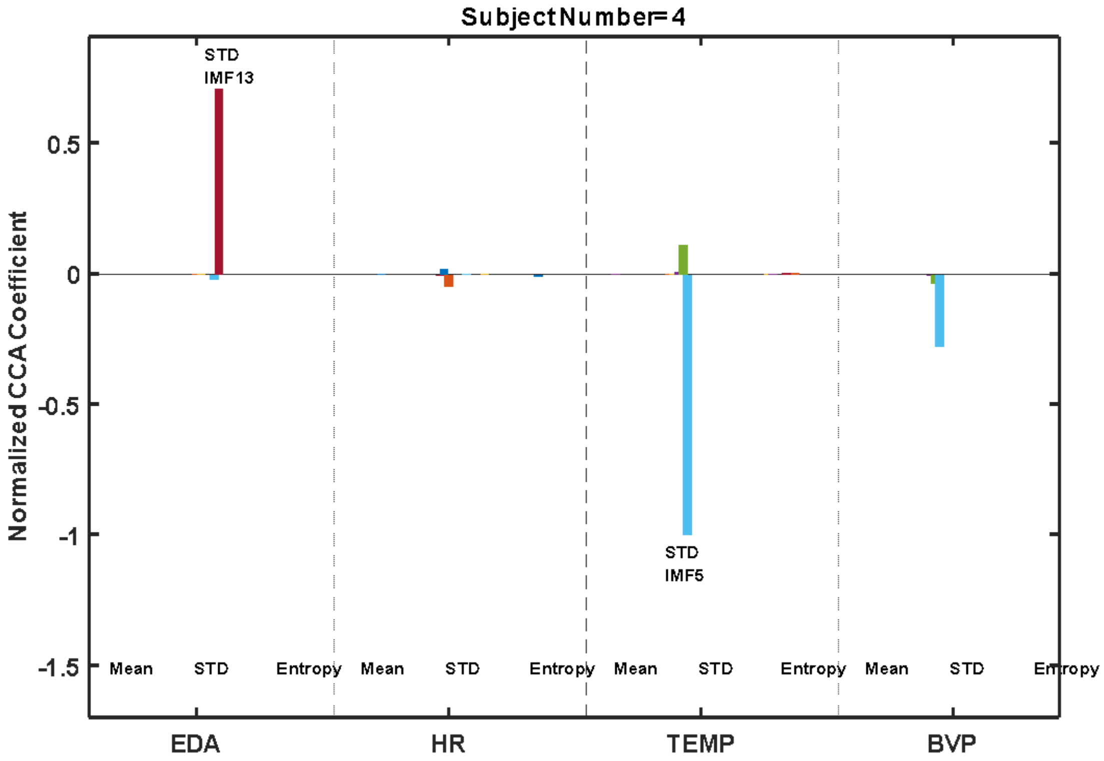
Subject Number=4
STD
IMF13
STD
IMF5
Normalized CCA Coefficient
0.5
0
-0.5
-1
-1.5
Mean STD Entropy Mean STD Entropy Mean STD Entropy Mean STD Entropy
EDA
HR
TEMP
BVP

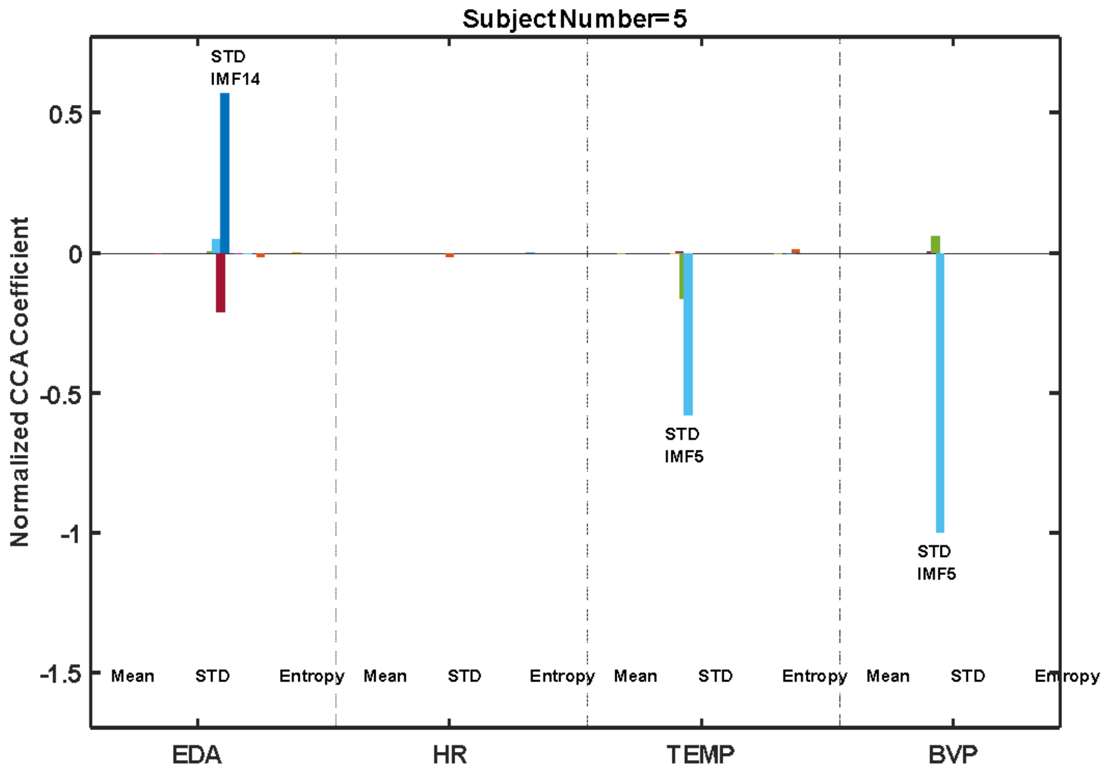
Subject Number=5
Normalized CCA Coefficient
0.5
0
-0.5
-1
-1.5
STD
IMF14
STD
IMF5
STD
IMF5
Mean
STD
Entropy
Mean
STD
Entropy
Mean
STD
Entropy
Mean
STD
Entropy
EDA
HR
TEMP
BVP

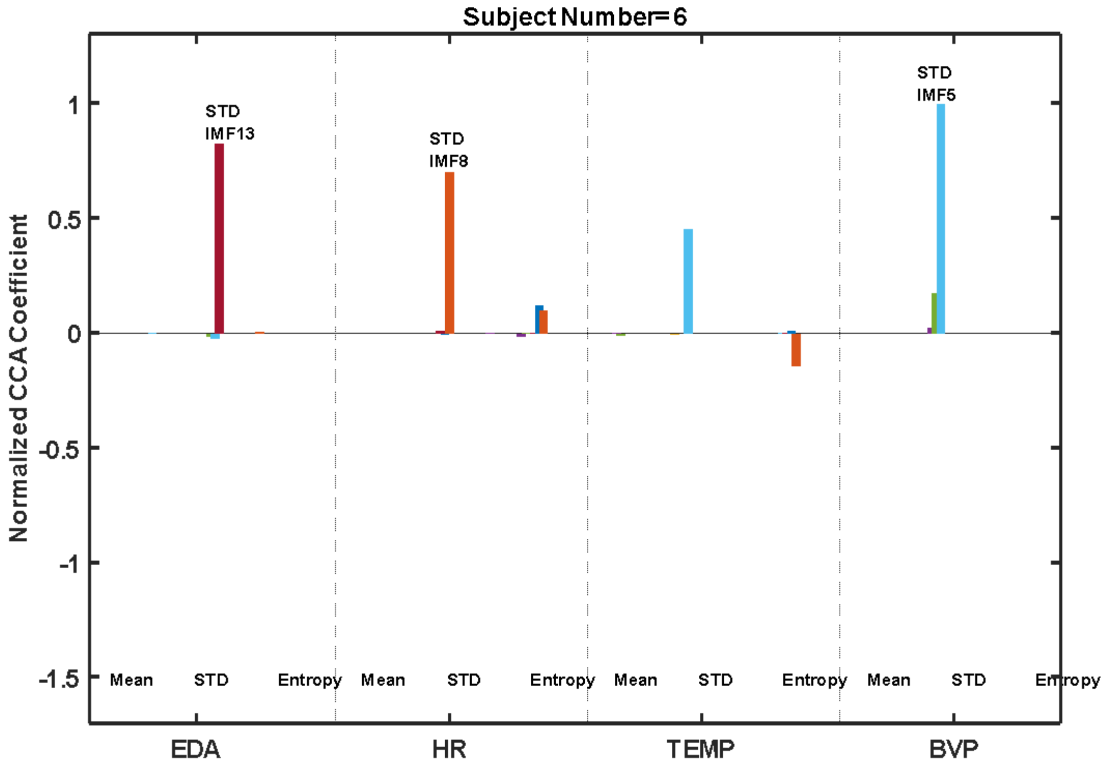
Subject Number=6
Normalized CCA Coefficient
1
0.5
0
-0.5
-1
-1.5
STD
IMF13
STD
IMF8
STD
IMF5
Mean
STD
Entropy
Mean
STD
Entropy
Mean
STD
Entropy
Mean
STD
Entropy
EDA
HR
TEMP
BVP

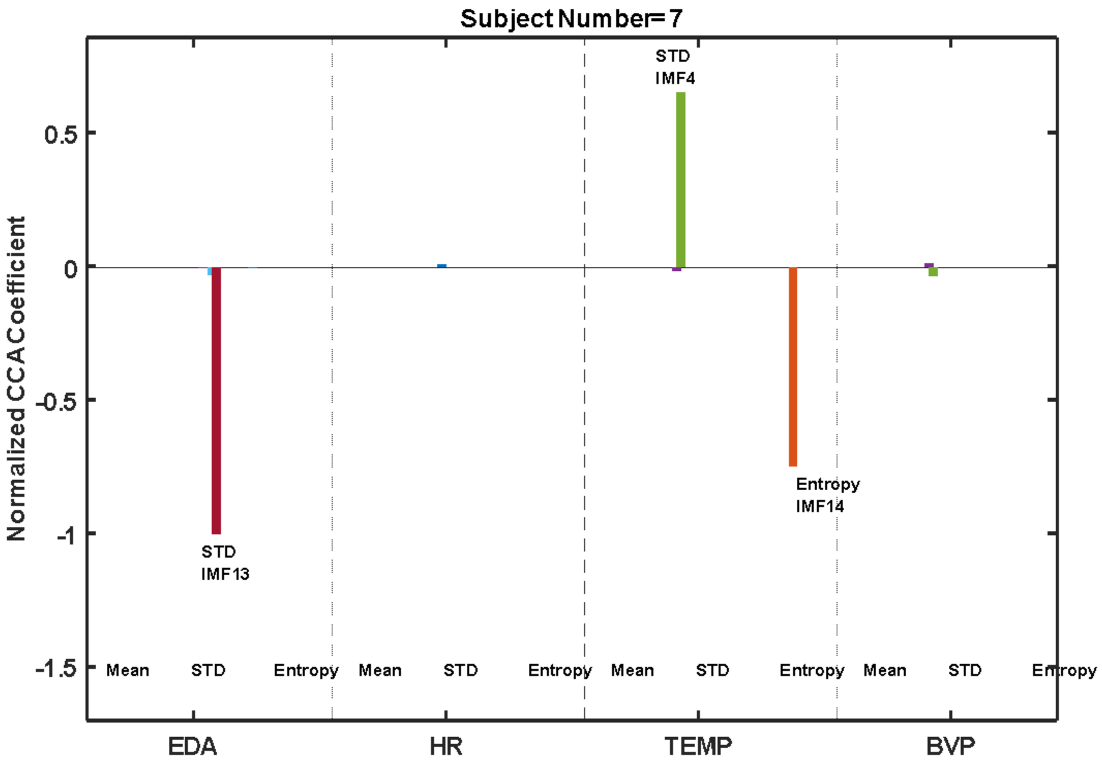
Subject Number=7
Normalized CCA Coefficient
0.5
0
-0.5
-1
-1.5
STD
IMF4
STD
IMF13
Entropy
IMF14
Mean
STD
Entropy
Mean
STD
Entropy
Mean
STD
Entropy
Mean
STD
Entropy
EDA
HR
TEMP
BVP

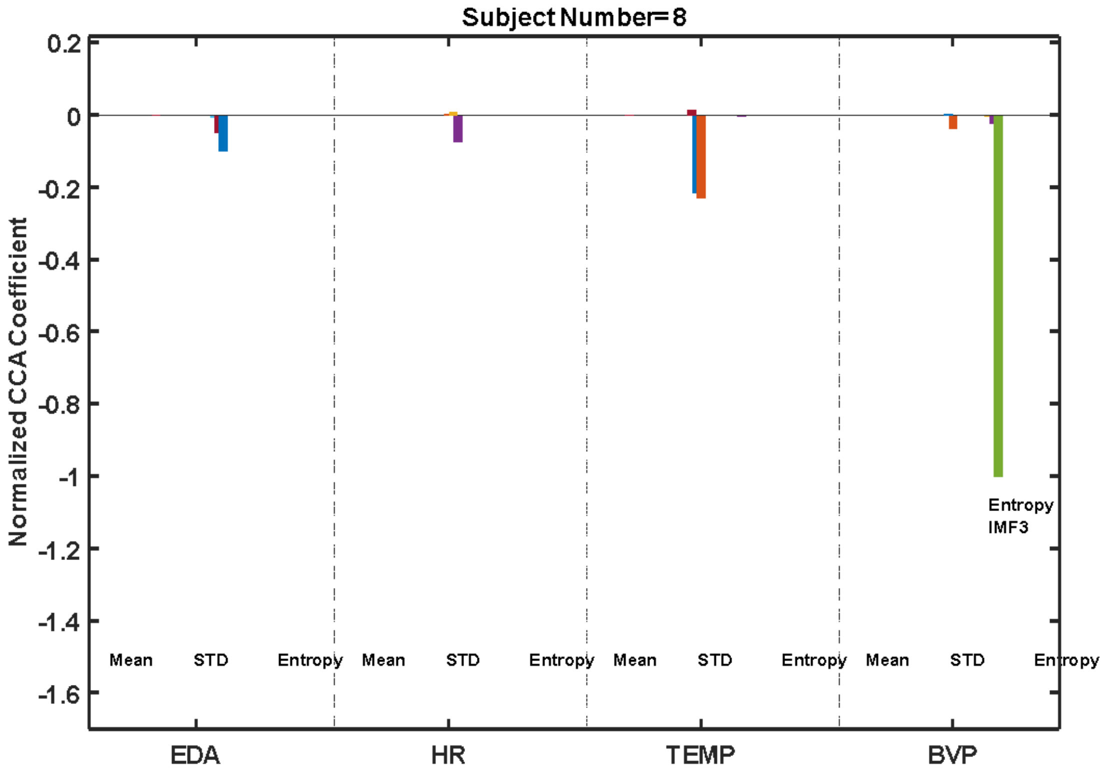
Subject Number=8
Normalized CCA Coefficient
0.2
0
-0.2
-0.4
-0.6
-0.8
-1
-1.2
-1.4
-1.6
Entropy
IMF3
Mean
STD
Entropy
Mean
STD
Entropy
Mean
STD
Entropy
Mean
STD
Entropy
EDA
HR
TEMP
BVP

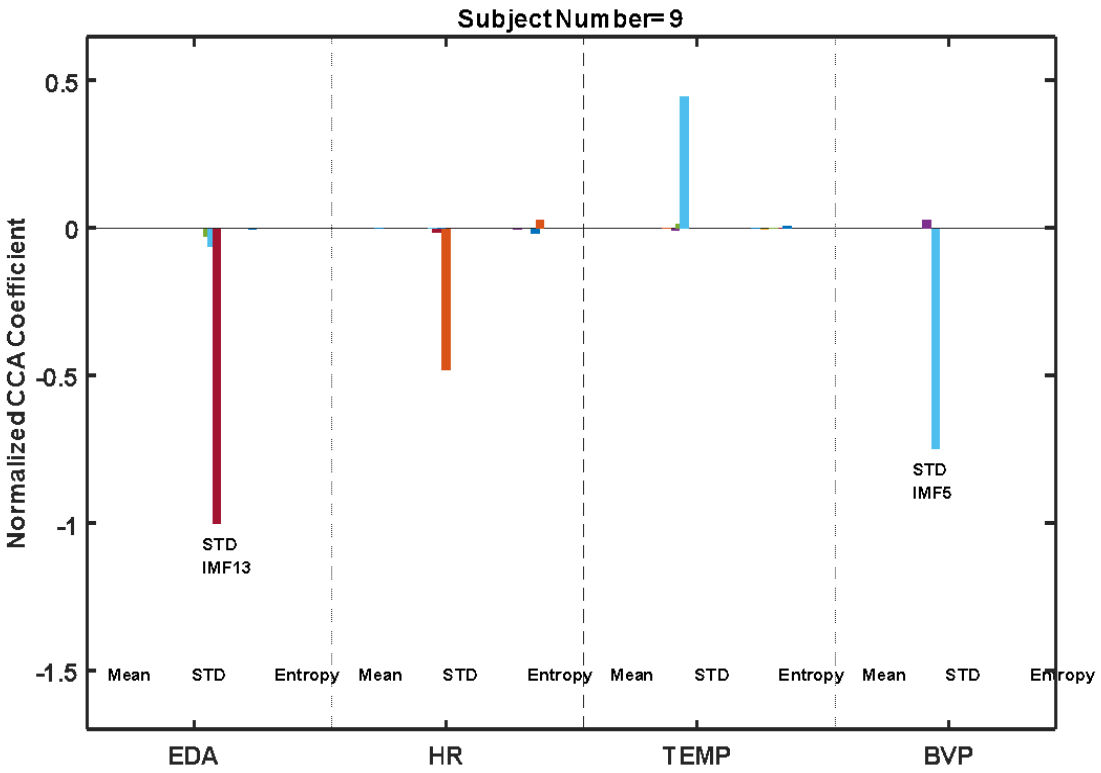
Subject Number=9
Normalized CCA Coefficient
0.5
0
-0.5
-1
-1.5
STD
IMF13
STD
IMF5
Mean
STD
Entropy
Mean
STD
Entropy
Mean
STD
Entropy
Mean
STD
Entropy
EDA
HR
TEMP
BVP

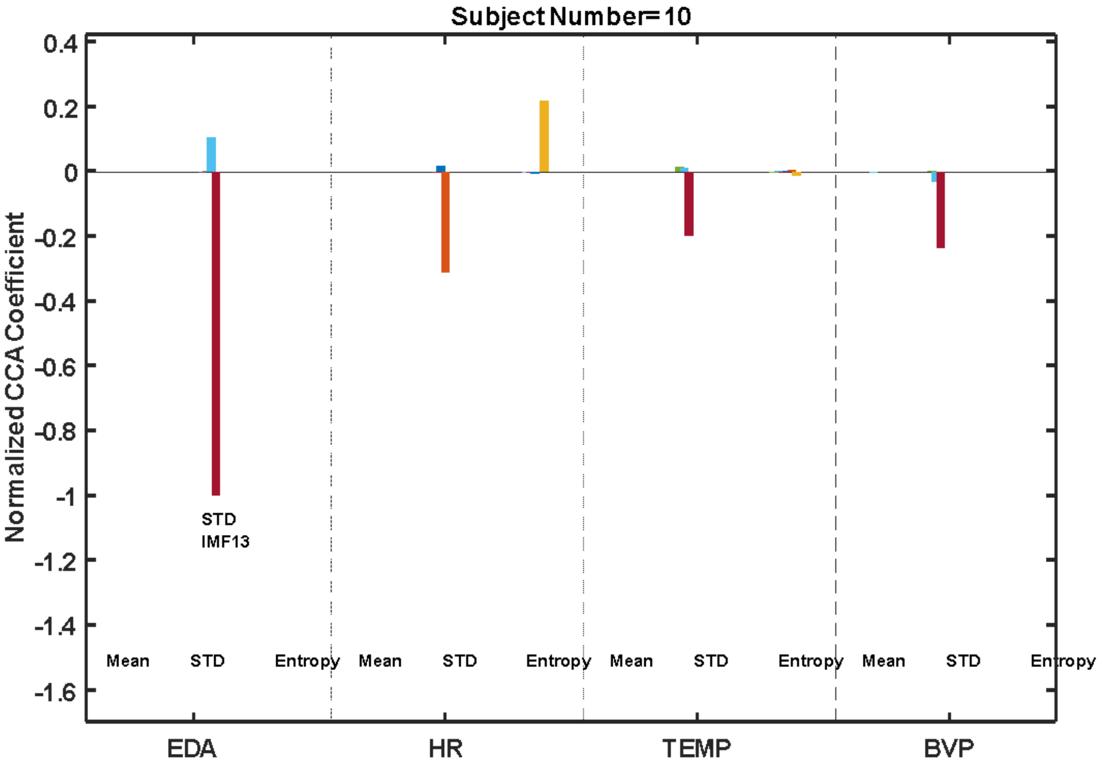
Subject Number=10
Normalized CCA Coefficient
0.4
0.2
0
-0.2
-0.4
-0.6
-0.8
-1
-1.2
-1.4
-1.6
STD
IMF13
Mean
STD
Entropy
Mean
STD
Entropy
Mean
STD
Entropy
Mean
STD
Entropy
EDA
HR
TEMP
BVP

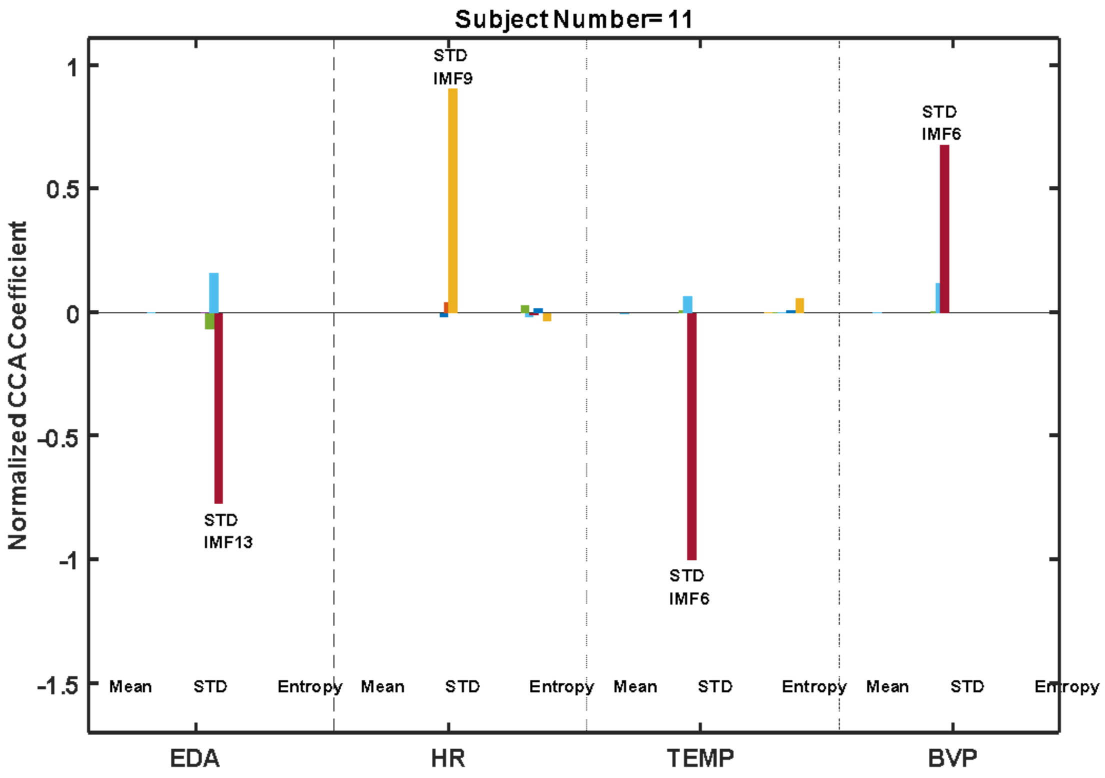

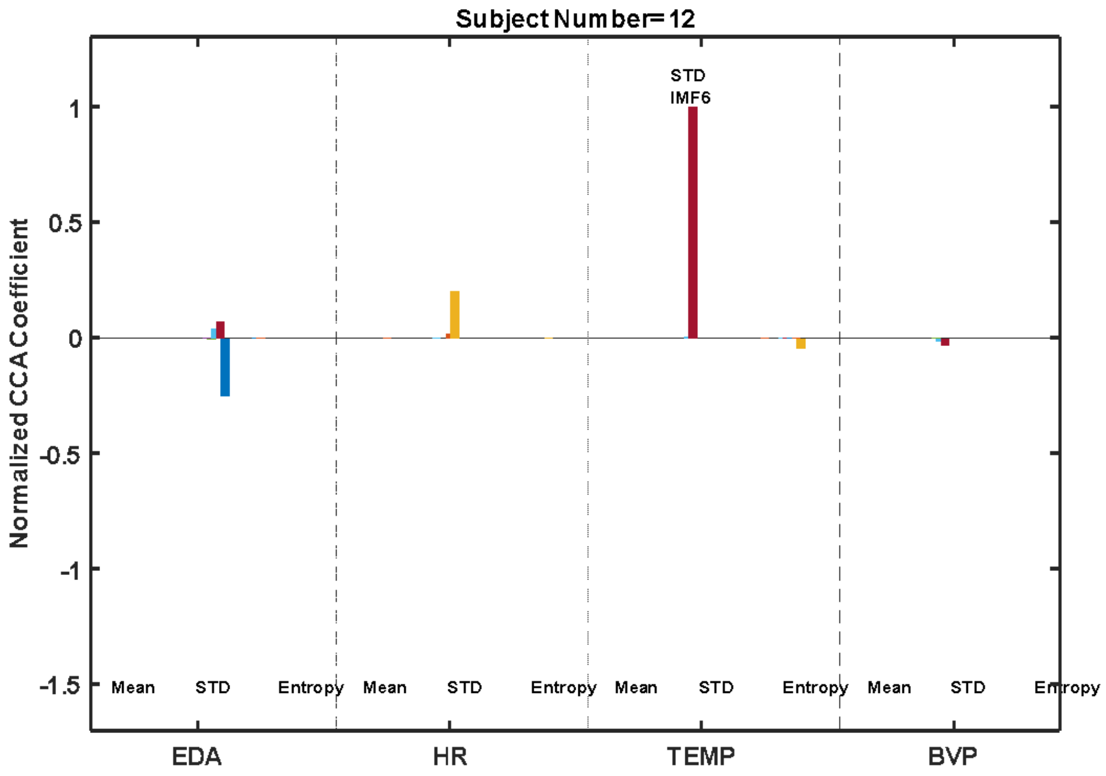

Figure S4. Normalized CCA coefficient weights for each feature obtained for each of 12 subjects.

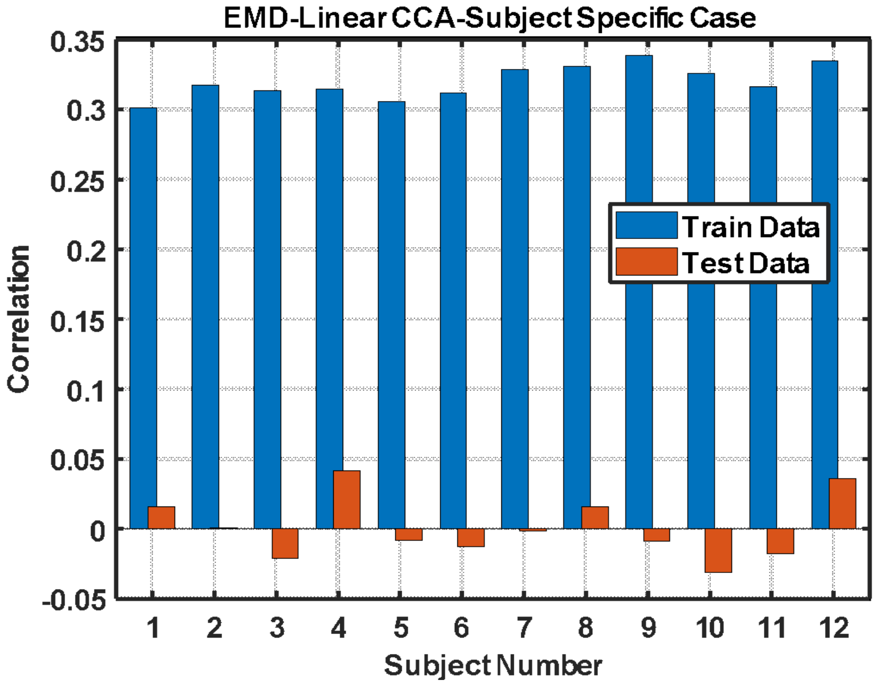

Figure S5. Correlation between a random vector with 2880 elements and extracted features of four sensor signals by subject-specific analysis. The subject number shows the experiment that 35 % of its 2880 samples were used as test data.

Table S1. P-value for computed correlation between test data of features and a random signal.

Signals	Subject Number											
	1	2	3	4	5	6	7	8	9	10	11	12
Random Signal	0.34	0.71	0.7	0.63	0.83	0.38	0.95	0.98	0.02	0.81	0.77	0.81
